# Group A Streptococcal Toxic Shock Syndrome after a Routine Gynecological Procedure

**DOI:** 10.1155/2021/9980015

**Published:** 2021-06-08

**Authors:** Kei Ameda, Yoko Yamada, Yuriko Uehara, Tamami Ohno, Mari Hoya, Hiroko Oda, Misako Mishima

**Affiliations:** Department of Obstetrics and Gynecology, Kawakita General Hospital, Tokyo, Japan

## Abstract

Streptococcal toxic shock syndrome (STSS) is a life-threatening illness mainly caused by invasive group A *Streptococcus* (GAS) infection. Herein, we report a case of a postmenopausal woman who developed STSS from an ascending vaginal GAS infection after cytocervical sampling. The patient complained of vaginal discharge, for which she underwent gynecological examination with vaginal sampling. The following day, there was onset of diarrhea and vomiting. After 7 days, she was admitted to our hospital with septic shock. Necrotizing enterocolitis was suspected and surgical intervention was performed; however, the patient was diagnosed with primary peritonitis and antibiotics were initiated. On day 2, GAS was suspected by blood cultures, and antibiotics were changed in consideration of STSS. On day 4, GAS was confirmed in blood, ascitic fluid, and vaginal swab specimens, and STSS caused by an ascending vaginal GAS infection was diagnosed. This case report indicates that STSS could occur following cytocervical sampling for vaginal discharge. If a woman has unexplained septic shock, especially with gastroenteritis symptoms, STSS should be considered as a differential diagnosis.

## 1. Introduction

Streptococcal toxic shock syndrome (STSS) is a critical illness caused by group A *Streptococcus* (GAS) and can rapidly lead to fatal multiorgan failure [1]. STSS is characterized by the isolation of GAS from sterile tissues, hypotension, and multiorgan involvement, as indicated by two or more of the following features: renal impairment, coagulopathy, liver involvement, acute respiratory distress syndrome, erythematous macular rashes, and soft-tissue necrosis [2] (Table 1). STSS has a high mortality rate [3]; for example, in the United States, the mortality rate of STSS was 10.7–12.0% between 2005 and 2012 [4]. Recently, the number of STSS cases has increased worldwide [4, 5]. Therefore, STSS is a critical disease that may be encountered more frequently in future clinical practice.

GAS is often found in the nasopharynx and skin [6]. Moreover, GAS colonizes the vagina, especially in women with lactational amenorrhea, menopausal women, and prepubescent girls [7–9], and vaginitis caused by GAS can progress to STSS [10]. Few studies reported that STSS caused by an ascending vaginal GAS infection may develop after interventional procedures, such as endometrial biopsy, intrauterine contraceptive device insertion, or hysteroscopy [11–15]. However, cytocervical sampling has not yet been recognized as a predisposing cause of STSS. Herein, we report a case of GAS-induced STSS after cytocervical sampling.

## 2. Case Presentation

A 51-year-old primipara postmenopausal woman presented to a local gynecology clinic with abnormal vaginal discharge and vulvar itching. She had no significant medical or surgical history. She underwent gynecological examination with vaginal discharge and cytocervical sampling as well as a transvaginal ultrasound examination. The following day, she experienced fever, diarrhea, and vomiting and went to see her local physician and was diagnosed with gastroenteritis. However, her symptoms persisted and she could hardly eat or drink. She had no history of recent consumption of raw food. Additionally, she developed weakness and difficulty in walking. She was then transported to our hospital 7 days after the initial gynecological examination. On arrival, her vital signs were as follows: blood pressure, 69/49 mmHg; respiratory rate, 25 breaths/min; heart rate, 62 beats/min; temperature, 37.7°C; oxygen saturation, 93%; and Glasgow coma scale score, 15. Physical examination revealed marked lower abdominal tenderness with muscle guarding. No erythematous macular rash was observed. Laboratory investigations showed an elevated white blood cell count (12,100/*μ*L) and C-reactive protein level (31.4 mg/dL) (Table 2). Transabdominal ultrasound and computed tomography showed intestinal edema, intestinal dilatation, and a large amount of ascites. Intravenous fluids were administered to restore the blood pressure to 92/74 mmHg. An initial diagnosis of gastroenteritis and dehydration was given.

Nine hours after admission, she lost consciousness and her blood pressure dropped again. Necrotizing enterocolitis and septic shock were suspected due to the patient's rapidly deteriorating clinical course, for which she underwent an exploratory laparotomy. This revealed >2,000 mL of milky white purulent ascites in the peritoneal cavity, without any signs of intestinal necrosis or other abdominal infection sources. Based on the intraoperative findings, the patient was diagnosed with primary peritonitis. After complete drainage and washing of the abdominal cavity, she was intubated and transferred to the intensive care unit. Empirical therapy with meropenem (1 g every 12 h) was initiated. Fluids, noradrenaline, immunoglobulins, and dopamine were administered intravenously for the treatment of severe septic shock. Laboratory investigations performed immediately after the shock and the surgery showed a sudden drop in platelet count (10.2 × 10^4^/*μ*L), a remarkably raised fibrin degradation products (FDP) (96.9 *μ*g/mL), and prolongation of prothrombin time and international normalized ratio (PT-INR) (1.43); hence, disseminated intravascular coagulation (DIC) was diagnosed because of the coagulopathy and administered thrombomodulin alpha and fresh frozen plasma (FFP) for the treatment of DIC.

On postoperative day 1, the patient developed acute kidney injury and underwent continuous hemodiafiltration and polymyxin B-immobilized column direct hemoperfusion. On day 2, we used gram staining, and gram-positive streptococci were isolated from all day 1 blood cultures, leading to the suspicion of GAS infection. Therefore, a rapid antigen detection test (RADT; Nippon Becton Dickinson and Company, Ltd.) was performed for blood, ascitic fluid, and vaginal swab specimens, to confirm GAS. Of note, this kit is usually used for throat swab specimens; however, all specimens were positive for GAS. Furthermore, the presence of hemolytic streptococcus was observed in the blood agar medium. Biochemical tests using reagents were used for detection of the streptococcus by immune serogroups, in which latex particles were sensitized with polyclonal antibodies against streptococcal groups A, B, C, D, F, and G, and we determined that the streptococci observed were GAS. Additionally, all the cultivation results of the blood, ascitic fluid, and vaginal swab specimens showed the presence of GAS. The blood and ascites culture tests were consistent with the specimens used for RADT; however, a different specimen was used for the vaginal culture test. Additionally, throat and urinary cultures were negative for GAS. The patient and her family had no history of throat infection. Then, antibiotic sensitivity tests were performed to identify antibiotic susceptibility and GAS was identified to be susceptible to ampicillin.

**Figure 1:**
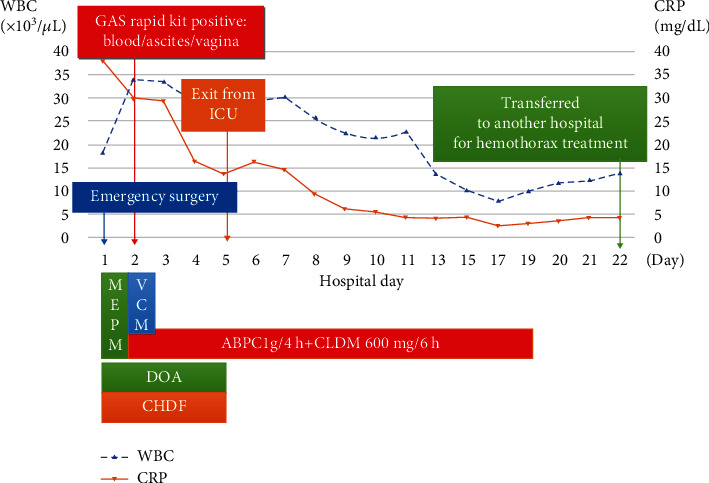
Course of inpatient treatment and clinical and laboratory parameters. WBC: white blood cells; CRP: C-reactive protein; GAS: group A *Streptococcus*; ICU: intensive care unit; MEPM: meropenem; VCM: vancomycin; ABPC: ampicillin; CLDM: clindamycin; DOA: dopamine; CHDF: continuous hemodiafiltration.

The patient was diagnosed with GAS-induced primary peritonitis and STSS caused by an ascending vaginal infection because we found GAS from a normally sterile site, hypotension, renal impairment, and DIC. The antibiotic regimen was changed to ampicillin (ABPC) (1 g every 4 h) and clindamycin (CLDM) (600 mg every 6 h) as first-line antibiotic choices for STSS; the patient improved and was extubated on day 5. After extubation, the patient had persistent respiratory failure and bilateral pleural effusions. On postoperative day 17, pleural effusion drainage was performed to improve respiratory status; it was a hemoperitoneal effusion. We investigated the cause of the hemoptysis and found hyperprolongation of PT-INR; thus, vitamin K2 and tranexamic acid were administered. On day 21, drainage was ineffective and her respiratory condition worsened; hence, she was transferred to another hospital for specialized management, where she recovered and was discharged without complications on day 66.  Figure 1 illustrates the patient's hospital treatment course and her clinical and laboratory parameters.

Patient data was anonymized, and the patient provided consent for publication of this case report.

## 3. Discussion

The patient in this study initially presented with a chief complaint of abnormal vaginal discharge and vulvar itching and was later found to have GAS vaginitis. Several cases of STSS caused by an ascending vaginal GAS infection have been reported in women who have lactational amenorrhea or are postmenopausal [16, 17]. In general, GAS is frequently isolated from vaginal swabs in such groups as well as in prepubertal girls [7–9]. The increased risk of GAS in these patients may be due to their low serum estrogen levels, which reduces the glycogen concentrations in the vagina, resulting in vaginal atrophy and a predisposition to GAS infections [18, 19]. We evaluated GAS cases detected by examination of vaginal swab specimens from January 2016 to August 2019 in our hospital. Twenty-one patients tested positive for GAS. Among them, the detection frequencies in women with lactational amenorrhea, postmenopausal women, prepubescent girls, and others were five (24%), five (24%), four (19%), and six (29%), respectively. Women with low estrogen levels accounted for two-thirds of the GAS-positive patients. Additionally, GAS vaginitis is common in prepubescent girls; however, there are few reports of a GAS infection progressing to STSS in prepubescent girls. Although it is unclear why STSS is more common in adults than children, a previous report suggested that the development of STSS in GAS carriers is associated with the host immune response [20].

The patient developed abdominal pain and nausea a day after the cytocervical sampling, with no earlier symptoms indicating GAS infection, except for abnormal discharge and vulvar itching. Later, microbiological testing revealed that while sputum, urine, and stool cultures were negative for GAS, the vaginal, ascites, and blood cultures were positive. Hence, we considered that GAS-induced primary peritonitis and STSS were caused by an ascending vaginal infection. Regarding the cause of vaginal ascending infection, while the cervical examining and sampling procedure may have contributed to the vaginal ascending of the infection, the infection itself could have been already present prior to gynecological inspection. In this context, atrophic vaginitis is caused by low estrogen. In atrophic vaginitis, low estrogen leads to thinning of the vaginal wall, decreased glycogen levels, decreased lactobacilli, and increased vaginal pH. This makes it easier for bacteria to multiply in the vagina and the vaginal walls to be damaged. In the present case, vaginal GAS infection was also caused by estrogen loss due to menopause. The weak vaginal wall, combined with gynecological examinations, may have contributed to the ascending infection, leading to STSS.

STSS is considered a rare disease; however, studies have reported an increasing incidence of STSS [4, 5, 21]. Thus, STSS is a critical disease that may be encountered more frequently in future clinical practice. STSS sporadically presents as primary peritonitis, making it difficult to diagnose in the early stages [22–25]. There are several case reports of primary GAS peritonitis in which fever, diarrhea, and vomiting were the first symptoms leading to the suspicion of gastroenteritis [22–25]. Moreover, in these cases, computed tomography findings showed only nonspecific intestinal edema and ascites. Therefore, early diagnosis of primary GAS peritonitis is difficult because of the lack of characteristic findings from physical examinations and imaging studies. If a woman has unexplained septic shock especially with gastroenteritis symptoms, STSS should be listed as a differential diagnosis. Particular attention should be paid in patients with lactational amenorrhea, postmenopausal women, or prepubescent girls or if there are gastrointestinal symptoms and abnormal discharge or a recent gynecological examination. The aforementioned risk factors should be checked during the medical interview.

Moreover, vaginal culture tests should be done as part of investigating the source of infection. The simple gram staining of the vaginal smear can confirm the presence of gram-positive cocci (GPC). If GPC is positive, species identification of GPC can lead to early diagnosis of STSS. The first choice of treatment for STSS is ABPC and CLDM.

In this case, GAS was found to be susceptible to ABPC, and we administered a high dose of ABPC. CLDM was used in combination because it inhibits the production of exotoxins and there is evidence that it improves prognosis [26]. Prompt initiation of ABPC and CLDM as first-line antibiotic choices for STSS is beneficial in improving patient prognosis. In contrast, CLDM-resistant GAS is on the rise in Japan; hence, we need to be careful [27].

## Figures and Tables

**Table 1:** The criteria of streptococcal toxic shock syndrome diagnosis [2].

Laboratory criteria	
(1) Isolation of group A *Streptococcus* from a normally sterile site.	

Clinical criteria	
(2) Hypotension	Systolic blood pressure ≤ 90 mmHg.
(3) Multiorgan involvement characterized by two or more of the following:	
(a) Renal impairment	Creatinine ≥ 2 mg/dL. In patients with preexisting renal disease, a greater than twofold elevation over the baseline level.
(b) Coagulopathy	Platelets ≤ 10 (×10^4^/*μ*L) or DIC.
(c) Liver involvement	Alanine aminotransferase, aspartate aminotransferase, or total bilirubin levels ≥ twice the upper limit of normal for the patient's age. In patients with preexisting liver disease, a greater than twofold increase over the baseline level.
(d) Acute respiratory distress syndrome	Defined by acute onset of diffuse pulmonary infiltrates and hypoxemia in the absence of cardiac failure or by evidence of diffuse capillary leak manifested by acute onset of generalized edema or pleural or peritoneal effusions with hypoalbuminemia.
(e) A generalized erythematous macular rash that may desquamate	
(f) Soft-tissue necrosis, including necrotizing fasciitis or myositis, or gangrene	

**Table 2:** Results of the laboratory investigation on admission.

Parameters	Result	Normal range
White blood cells (/*μ*L)	12,100	3,500–8,500
Red blood cells (×10^4^/*μ*L)	457	385–435
Hemoglobin (g/dL)	14	12.0–16.0
Platelets (×10^4^/*μ*L)	14.2	15.0–35.0
Albumin (g/dL)	1.28	3.8–5.3
BUN (mg/dL)	83.6	8.0-20.0
Creatinine (mg/dL)	3.1	0.4–0.8
Aspartate aminotransferase (IU/L)	96	7–38
Alanine aminotransferase (IU/L)	30	4–36
Lactate dehydrogenase (IU/L)	801	106–220
Creatinine phosphokinase (U/L)	1,027	45–165
C-reactive protein (mg/dL)	31.04	0–0.3
Prothrombin time (sec)	12.9	10–13
Activated partial thrombin time (sec)	27.4	25–40

## Data Availability

No data were used to support this study.

## References

[1] Wahab A., Nasir B. (2018). Streptococcal toxic shock syndrome with primary group A streptococcus peritonitis in a healthy female. *Journal of Community Hospital Internal Medicine Perspectives*.

[2] *Streptococcal Toxic Shock Syndrome | 2010 Case Definition n.d.*.

[3] Linnér A., Darenberg J., Sjölin J., Henriques-Normark B., Norrby-Teglund A. (2014). Clinical efficacy of polyspecific intravenous immunoglobulin therapy in patients with streptococcal toxic shock syndrome: a comparative observational study. *Clinical Infectious Diseases*.

[4] Nelson G. E., Pondo T., Toews K.-A. (2016). Epidemiology of invasive group A streptococcal infections in the United States, 2005–2012. *Clinical Infectious Diseases*.

[5] Rudolph K., Bruce M., Bruden D. (2016). Epidemiology of invasive group A streptococcal disease in Alaska, 2001 to 2013. *Journal of Clinical Microbiology*.

[6] A Group A Streptococcal Infection | CDC (2019). https://www.cdc.gov/groupastrep/diseases-public/scarlet-fever.html.

[7] Stricker T., Navratil F., Sennhauser F. H. (2003). Vulvovaginitis in prepubertal girls. *Archives of Disease in Childhood*.

[8] Morris C. A. (1971). Seasonal variation of streptococcal vulvo-vaginitis in an urban community. *Journal of Clinical Pathology*.

[9] Verstraelen H., Verhelst R., Vaneechoutte M., Temmerman M. (2011). Group A streptococcal vaginitis: an unrecognized cause of vaginal symptoms in adult women. *Archives of Gynecology and Obstetrics*.

[10] Stevens D. L., Tanner M. H., Winship J. (1989). Severe group A streptococcal infections associated with a toxic shock-like syndrome and scarlet fever toxin A. *The New England Journal of Medicine*.

[11] Cho E. E., Fernando D. (2013). Fatal streptococcal toxic shock syndrome from an intrauterine device. *The Journal of Emergency Medicine*.

[12] Venkataramanasetty R., Aburawi A., Phillip H. (2009). Streptococcal toxic shock syndrome following insertion of an intrauterine device – a case report. *The European Journal of Contraception & Reproductive Health Care*.

[13] Marshall B. R., Hepler J. K., Jinguji M. S. (1973). Fatal *Streptococcus pyogenes* septicemia associated with an intrauterine device. *Obstetrics and Gynecology*.

[14] Gisser J. M., Fields M. C., Pick N., Moses A. E., Srugo I. (2002). Invasive group A streptococcus associated with an intrauterine device and oral sex. *Sexually Transmitted Diseases*.

[15] Mourton S., Rich W. (2006). Group A streptococcal toxic shock syndrome after an office endometrial biopsy: a case report. *The Journal of Reproductive Medicine*.

[16] Iwata Y., Iwase S. (2017). Group A streptococcal peritonitis and toxic shock syndrome in a postmenopausal woman. *Internal Medicine*.

[17] Hikone M., Kobayashi K., Washino T. (2015). Streptococcal toxic shock syndrome secondary to group A streptococcus vaginitis. *Journal of Infection and Chemotherapy*.

[18] Meltzer M. C., Schwebke J. R. (2008). Lactational amenorrhea as a risk factor for group a streptococcal vaginitis. *Clinical Infectious Diseases*.

[19] Rahangdale L., Lacy J., Hillard P. A. (2008). Group A streptococcus vulvovaginitis in breastfeeding women. *American Journal of Obstetrics and Gynecology*.

[20] Stevens D. L. (1992). Invasive group A streptococcus infections. *Clinical Infectious Diseases*.

[21] Henningham A., Barnett T. C., Maamary P. G., Walker M. J. (2012). Pathogenesis of group A streptococcal infections. *Discovery Medicine*.

[22] Graham J. C., Moss P. J., McKendrick M. W. (1995). Primary group A streptococcal peritonitis. *Scandinavian Journal of Infectious Diseases*.

[23] Haap M., Haas C. S., Teichmann R., Horger M., Raible A., Lamprecht G. (2010). Mystery or misery? Primary group A streptococcal peritonitis in women: case report. *American Journal of Critical Care*.

[24] Kanetake K., Hayashi M., Hino A. (2004). Primary peritonitis associated with streptococcal toxic shock-like syndrome: report of a case. *Surgery Today*.

[25] Monneuse O., Tissot E., Gruner L. (2010). Diagnosis and treatment of spontaneous group A streptococcal peritonitis. *The British Journal of Surgery*.

[26] Mulla Z. D., Leaverton P. E., Wiersma S. T. (2003). Invasive group A streptococcal infections in Florida. *Southern Medical Journal*.

[27] Sakata H. (2015). The change of macrolide resistance rates in group A streptococcus isolates from children between 2002 and 2013 in Asahikawa city. *Journal of Infection and Chemotherapy*.

